# Phosgene synthesis catalysis: reaction kinetics and adsorption characteristics over Norit RX3 Extra activated carbon formulation†

**DOI:** 10.1039/d5ra04045k

**Published:** 2025-07-16

**Authors:** Rory Hughes, David Lennon

**Affiliations:** a School of Chemistry, University of Glasgow Joseph Black Building Glasgow G12 8QQ UK David.Lennon@glasgow.ac.uk +44-141-330-4372

## Abstract

The authors have recently refined a model for phosgene synthesis over industrial grade activated carbons that involves two classes of active sites: type-I and type-II. This article looks to further validate the model by examining kinetic aspects of the reaction. The work focuses on a single formulation of activated carbon, Norit RX3 Extra, and extends the applicability of the previous analysis undertaken on the Donau Supersorbon K40 formulation of activated carbon; both materials are representative industrial grade catalysts active for phosgene synthesis. The orders of this reaction, with respect to reagents and products CO, Cl_2_ and COCl_2_ are 1.04 ± 0.02, 0.46 ± 0.02, 0.04 ± 0.01 respectively. These findings reproduce the observations over Donau Supersorbon K40 and further validate the proposed reaction model. However, non-competitive adsorption studies over fresh catalyst reveal the following order of adsorption coefficients (*K*): *K*_COCl_2__ > *K*_Cl_2__ ≫ *K*_CO_. This contrasts with the studies over Donau Supersorbon K40, suggesting a different distribution of active sites. Studies that regenerate the catalyst and re-adsorb chlorine show the concentrations of type-I and type-II Cl_2_ adsorption sites are 3.2 and 0.32 mMol Cl_2_ per g_(cat)_, respectively; the retention on type I sites being 3.5 times greater for the Norit material than is observed for the Donau sample. Mass balance profiles endorse these findings. Temperature-programmed breakthrough measurements are interpreted as indicating a degree of surface etching of the carbonaceous substrate. Whilst this work reports some distinct differences between the two catalysts, the recently amended phosgene synthesis reaction model is validated over Norit RX3 Extra, enhancing the model's credentials as being representative for phosgene synthesis over activated carbon formulations.

## Abstract 2

A commercial activated carbon (Norit RX3 Extra) is examined with respect to phosgene synthesis *via* rate law determination and non-competitive adsorption measurements. A proposed reaction model is validated through these measurements.

## Graphical abstract

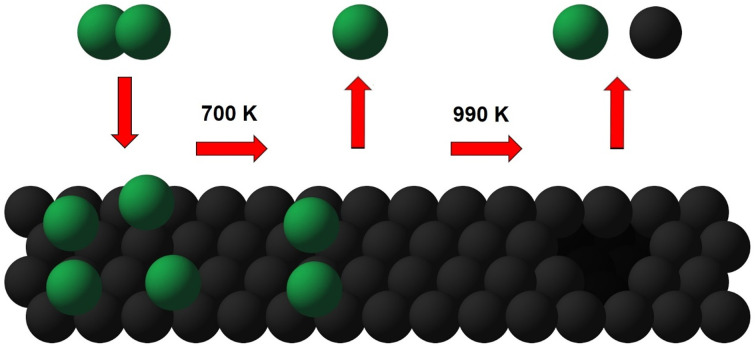

## 1. Introduction

Phosgene (COCl_2_) is an important industrial reagent, produced on a global scale of 12 Mt annually.^1^ This value is anticipated to exceed 18 Mt by 2030.^2^ While phosgene has niche uses in the production of pharmaceuticals, agrochemicals and dyestuffs,^3–5^ it is estimated that 95% of the global use of phosgene is in the production of polyurethanes and polycarbonates.^2^ For this use, phosgene is almost exclusively produced *via* the gas phase combination of carbon monoxide (CO) and chlorine (Cl_2_) over an activated carbon catalyst (eqn (1)).^6^ This process is exothermic, (Δ*H* = −107.6 kJ mol^−1^) and, as such, is associated with high operating temperatures, which can exceed 773 K.^3,7–11^

1 CO + Cl_2_ ⇌ COCl_2_

Despite the phosgene synthesis process being commercially highly valuable, there are surprisingly few studies of this reaction in the literature.^12^ This is likely due to the operational^13^ and occupational^14^ hazards associated with the reagents and products. In addition, the electronic structure of activated carbons^15^ make analysis of the catalyst difficult using traditional optical spectroscopy. Furthermore, different formulations of activated carbon possess different amounts of surface functionalities.^16^ These functionalities, and the proportions of them possessed by a given formulation, can significantly affect the properties of an activated carbon. For example, activated carbons are known to have an acidic, neutral or basic character depending on the amount and ratio of heteroatoms, such as oxygen or nitrogen.^16,17^ Due to the aforementioned electronic structure issues, determining the presence and number of individual surface functionalities (*e.g.* carboxyl, carbonyl and phenol groups) can prove challenging. Recent advancements in the analysis of highly carbonaceous materials have been driven by the development of temperature programmed analysis.^18^

**Scheme 1:**
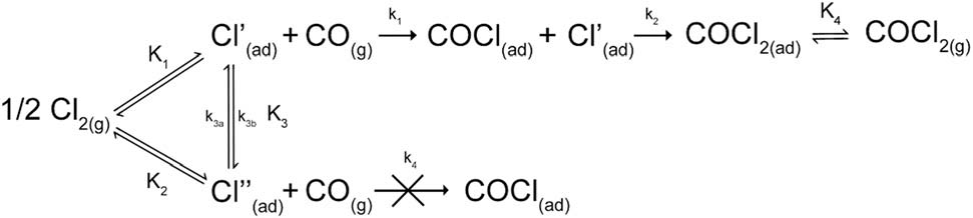
A reaction model for phosgene synthesis from carbon monoxide and chlorine over activated carbon. Reproduced with permission from ref. 33.

While there are numerous functionalities present on a typical activated carbon, only a handful have been shown to interact with chlorine,^19–24^ namely hydrides, hydroxyls, carboxyls, olefinic bonds and graphitic structures. Possibly reflecting this range of potential active sites, differences in the mechanism for phosgene synthesis catalysis over activated carbon exist.^3,22–30^ Whilst it is generally accepted that chlorine adsorption is the key to phosgene synthesis,^22–24,30,31^ there is some disparity on the mechanism by which the reaction occurs. Some publications favour a Langmuir–Hinshelwood type mechanism between CO and Cl_2_.^3,25–29^ On the other hand, the surface bound chlorine interacting with ballistic CO in an Eley–Rideal type mechanism was first proposed by Satterfield,^32^ with further experimental weight given by Gupta and co-workers.^22–24^ Two recent publications from this laboratory looking at phosgene synthesis over two different industrial grade catalysts, Donau Supersorbon K40 (ref. 30) and Norit RX3 Extra,^33^ have refined a reaction model that defines roles for two classes of active site: type-I and type-II (Scheme 1). Chlorine atoms are adsorbed at both sites but only the type-I site supports phosgene formation; the type-II site is inactive to phosgene production under the conditions studied.^33^ The model, based on a range of experimental data that includes mass balance profiles, combines an Eley–Rideal stage (*k*_1_) with a Langmuir–Hinshelwood stage (*k*_2_). This scenario seemingly unites the range of surface interaction models previously considered.

Scheme 1 was derived from experimentation using the Norit RX3 Extra formulation of activated carbon^33^ and results therein compared to results produced over the Donau Supersorbon K40 formulation of activated carbon.^30^ The focus of this publication is to further interrogate the reaction model using kinetic and mechanistic insight gleaned from rate order and non-competitive adsorption studies over Norit RX3 Extra. Following the approach of Weller,^26^ reaction orders are combined to define the rate law, a fundamental parameter of reaction kinetics. Comparisons of outcomes for the two commercial grade activated carbons scrutinise the generic nature of Scheme 1 to account for phosgene synthesis over industrial grade activated carbon formulations.

## 2. Experimental

The experimental apparatus has been comprehensively described elsewhere.^13^ and its adoption is briefly outlined here. The flow of reagents (CO, BOC, CP grade and Cl_2_, CK Isotopes, 99.9% purity), diluent (N_2_, BOC, 99.998% purity) and phosgene (BOC, 10% COCl_2_ in He) were metered by mass flow controllers (Hastings, CO, N_2_ COCl_2_ – HFC-202, Cl_2_ – HFC-302). The diluent N_2_ was partitioned as a flow pre-reactor with a secondary flow post-reactor, with the partitioning maintaining a suitable residence time of the gasses in the reactor, whilst ensuring the products remain in the gas phase for analysis.

The gasses were contained by 1/8-inch Swagelok stainless steel tubing. The reactor was a quartz tube (outer diameter 6.35 mm) which contained 0.1250 g of catalyst (Norit RX3 Extra Activated Carbon, Merck, 901934-500G), ground to between 250 and 500 μm and filtered using sieves (Endcotts). Quartz wool (Elemental Microanalysis) plugs were used to hold the catalyst bed in place. The reactor was housed in a temperature-controlled oven (TF1 11/32/150, Carbolite Gero) and the catalyst was dried at 373 K for 16 hours prior to reaction in a 7 ml min^−1^ flow of nitrogen. A by-pass reactor was also connected which contained an equal volume of ground quartz, 0.4410 g, ground to the same size fraction (250–500 μm).

The gasses eluting from the reactor were analysed by a combination of infrared (IR, Is10, Nicolet) and ultraviolet/visible (UV/vis, UV-1800, Shimadzu) spectroscopy. The spectral acquisition time for the IR and UV/vis spectrometers were 60 seconds and 50 seconds, respectively. The gasses were neutralised post-analysis with a 2 molar sodium hydroxide chemical scrubber prior to extraction from the fume cupboard.

### 2.1. Reaction order

The exact details of the different procedures adopted for the different chemical species (CO, Cl_2_, and COCl_2_) will be considered individually. However, the general the procedure, which adopts the van't Hoff method^34^ (aka the isolation method) is as follows. The gas flow was allowed to pass through the by-pass reactor for 30 minutes at room temperature, by which point the spectroscopic signals had stabilised. Spectra were then taken of the reagent being studied. The values for this species represent the initial flow of said species entering the reactor, or *A*_0_. The flow of the gasses was then directed over the reactor containing the catalyst maintained at 323 K. The temperature of 323 K was chosen as previous studies^33^ have shown this system to be highly reactive, with full consumption of Cl_2_ achieved at 473 K. As such, to investigate intrinsic kinetics, this lower temperature was determined to be suitable. The values of phosgene produced was followed in 10 minute intervals for 60 minutes before the flow was returned to the bypass and the flow rates adjusted. The reaction system has previously been reported^33^ to readily achieve steady-state conditions. As such, the 4 replicate measurements taken at each time interval were averaged for a given flow rate, so that each data-point represents an average of 16 spectra, with the error bars representing the standard deviation of this average. The pre-reactor flow rate of N_2_ was adjusted to maintain a constant flow rate through the reactor.

The reaction order of CO was studied at 323 K by flowing Cl_2_ in a large excess (15 ml min^−1^) and varying the flow rate of CO between 2 and 6 ml min^−1^ in 1 ml min^−1^ intervals. Table S1† displays the varying flow rates used in this experiment.

The reaction order of Cl_2_ was obtained in a similar manner to that described for CO but, instead, the CO was fixed at 15 ml min^−1^ and the Cl_2_ flow rate varied. However, Cl_2_ flow rates between 2 and 6 ml min^−1^ and a total reactor flow rate of 59 ml min^−1^ resulted in a total reagent conversion of around 17.5%, which corresponds to an integral regime.^35^ Increasing the dilution flow, so that the total flow of gasses through the reactor was 69 ml min^−1^, and increasing the flow range of Cl_2_ to between 6 to 10 ml min^−1^, resulted in an average total reagent conversion of 9%. While this value is still on the high side, is has been shown previously that this system is free from any temperature or diffusion limitations when operating under comparable conditions.^13^ Table S2† shows the varying flow rates used in this experiment.

The order of COCl_2_ was obtained by holding both CO and Cl_2_ in a large excess (15 ml min^−1^), while varying the phosgene flow rate (diluted to 10% in helium) between 10 ml min^−1^ and 20 ml min^−1^ in 2.5 ml min^−1^ increments (actual COCl_2_ flow rate of between 1 ml min^−1^ and 2 ml min^−1^ in 0.25 ml min^−1^ increments). In addition to this, typical operation of the apparatus does not include flowing a stock of phosgene in addition to the two reagents and nitrogen.^13^ As such, the apparatus was modified to include an extra nitrogen line with a mass flow controller as shown in Fig. S1.† The total flow rate through the reactor was chosen to be 69 ml min^−1^ in a similar manner to the order dependence of Cl_2_ experiment. The flow rates used to determine the order dependence of COCl_2_ are presented in Table S3.†

### 2.2. Non-competitive adsorption (breakthrough measurements)

#### 2.2.1 Measurements at 323 K

The non-competitive adsorption measurements were performed at 323 K over a fresh catalyst for all three of the species investigated, CO, Cl_2_ and COCl_2_. The flow rate of CO was 5 ml min^−1^ with Cl_2_ and COCl_2_ being flowed at 4 ml min^−1^ (10% COCl_2_ in He total flow 40 ml min^−1^). The total flow through the reactor was maintained at 59 ml min^−1^ by utilising a N_2_ flow of either 54 ml min^−1^ (CO), 55 ml min^−1^ (Cl_2_) or 19 ml min^−1^ (COCl_2_). The gasses were allowed to flow over the bypass for 60 minutes, prior to the first sample being taken. The reaction was followed until it was determined that the value of chemical species had returned to baseline values, *i.e.*, the catalyst's capacity was reached. As a baseline measurement, the process was repeated over a reactor containing an equal volume of quartz, ground to the same size fraction (0.4410 g and 250 and 500 μm, respectively).

The adsorption capacity of the catalyst was determined for each species as shown in Fig. S2.† The area of the consumed molar flow rate was determined through integration. The integrated area determined over quartz was subtracted from the area found over the active catalyst as quartz has previously been shown to be inactive for adsorption of these species and COCl_2_ synthesis.^13,36^

#### 2.2.2 Thermal treatments

Chlorine breakthrough measurements on Donau Supersorbon K40,^30^ undertaken at 323 K after the chlorine saturated catalyst had been held in a nitrogen carrier flow for 2 h at 673 K indicated chlorine retention to involve low and higher energy adsorption sites. Indeed, these measurements led to the conceptualisation of type-I and type II chlorine adsorption sites, with 673 K representing an arbitrary divider (the highest operational temperature of the oven used for those measurements). On this basis, comparable measurements were applied to Norit RX3 Extra. However, in recognition that TGA studies of Norit RX3 Extra performed in an oxidising atmosphere (2% O_2_/Ar) showed bulk decomposition of the activated carbon to occur at temperatures > 873 K,^33^ the chlorine breakthrough measurements were undertaken after thermal ramping below (700 K) and above (990 K) the thermal decomposition onset temperature. Specifically, the chlorine saturated sample prepared at 323 K was heated to 700 K at a rate of 5 K min^−1^ while being purged with a 10 ml min^−1^ flow of nitrogen. The sample was then left to purge and cool to room temperature for 16 hours before a further non-competitive adsorption of chlorine measurement was taken. This process was again repeated on heating to 990 K, with the higher temperature providing an indication of catalyst form under conditions that could be encountered in the large-scale operation of the phosgene synthesis process.

## 3. Results and discussion

### 3.1. Reaction order

**Fig. 1:**
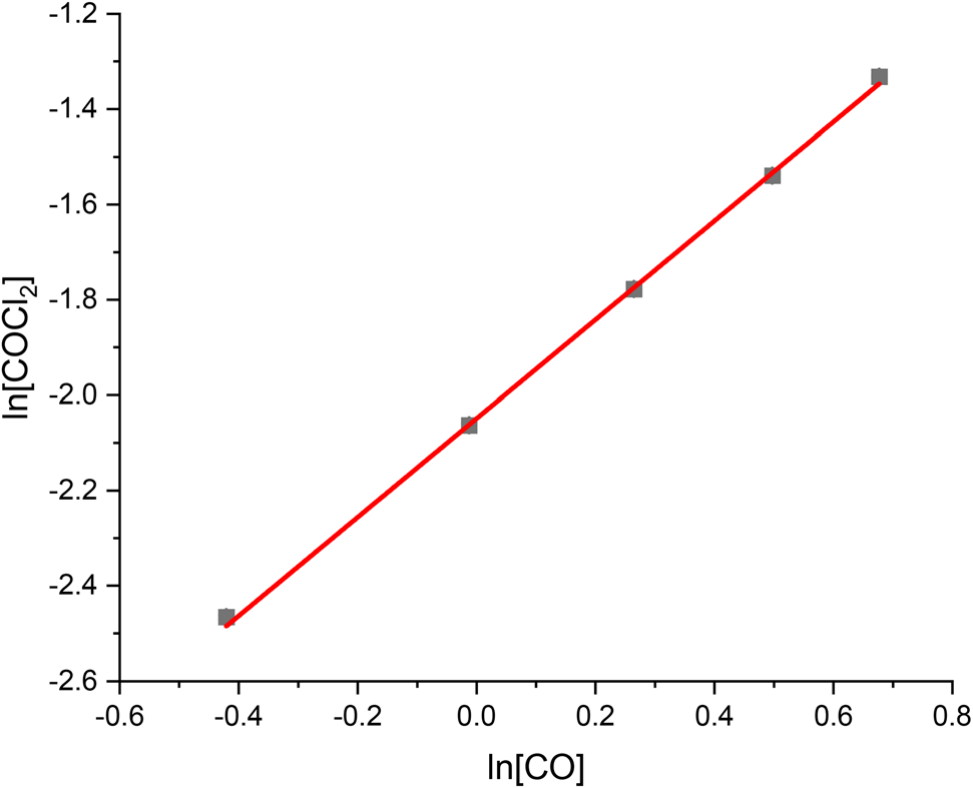
van't Hoff plot of the concentration dependence of CO for the reaction between CO and Cl_2_ over 0.1250 g of the Norit RX3 Extra formulation of activated carbon, ground to between 250 and 500 μm at 323 K. The order dependence was determined by fixing Cl_2_ at 15 ml min^−1^ and varying the flow rate CO between 2 ml min^−1^ and 6 ml min^−1^ in 1 ml min^−1^ steps, while varying the flow of the N_2_ diluent gas between 42 ml min^−1^ and 38 ml min^−1^ to maintain a total flow through the reactor of 59 ml min^−1^. The associated spectra are presented in Fig. S3.†

**Fig. 2:**
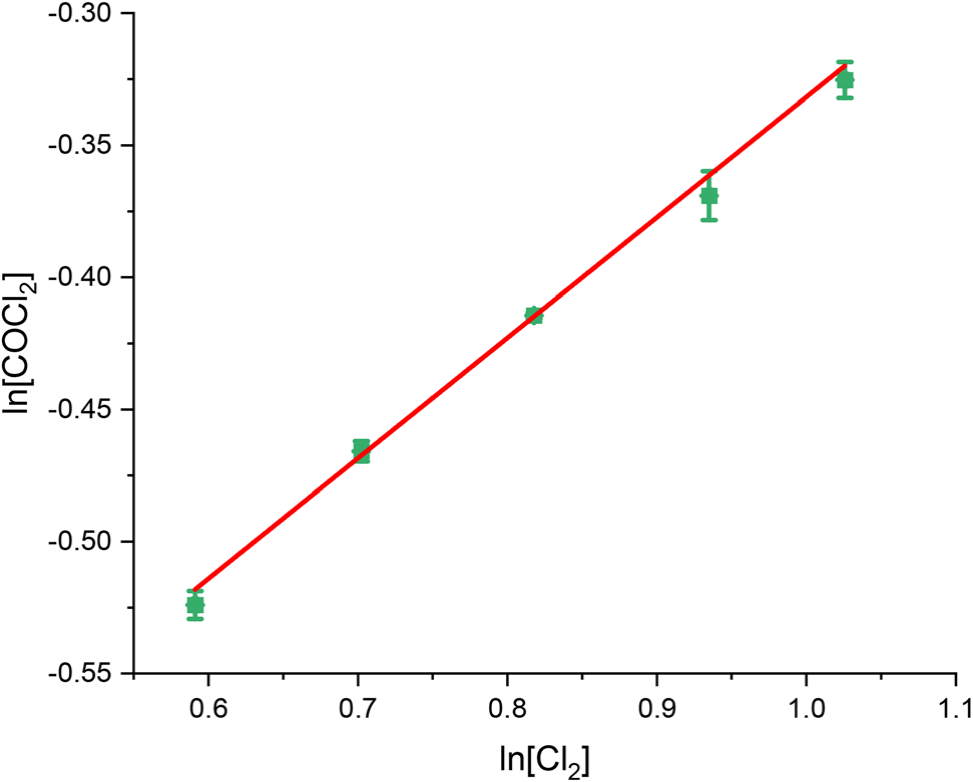
van't Hoff plot of the concentration dependence of Cl_2_ for the reaction between CO and Cl_2_ over 0.1250 g of the Norit RX3 Extra formulation of activated carbon, ground to between 250 and 500 μm at 323 K. The order dependence was determined by fixing CO at 15 ml min^−1^ and varying the flow rate CO between 6 ml min^−1^ and 10 ml min^−1^ in 1 ml min^−1^ steps, while varying the flow of the N_2_ diluent gas between 48 ml min^−1^ and 44 ml min^−1^ to maintain a total flow through the reactor of 69 ml min^−1^. The associated spectra are presented in Fig. S4.†

**Fig. 3:**
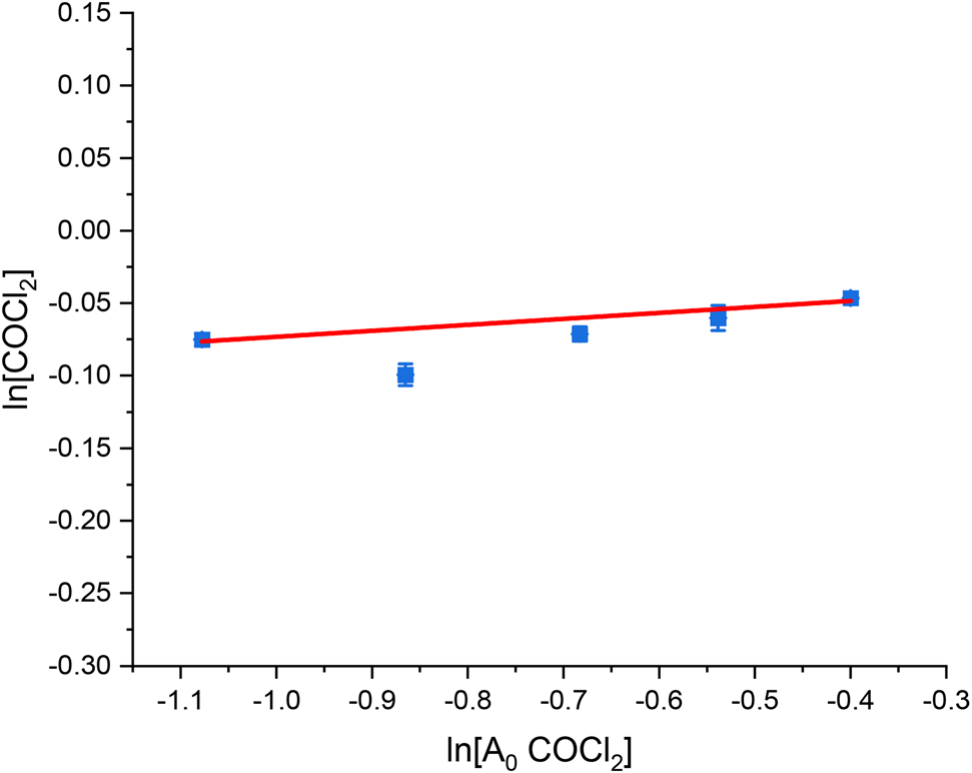
van't Hoff plot of the concentration dependence of COCl_2_ for the reaction between CO and Cl_2_ over 0.1250 g of the Norit RX3 Extra formulation of activated carbon, ground to between 250 and 500 μm at 323 K. The order dependence was determined by fixing CO and Cl_2_ at 15 ml min^−1^ and varying the flow rate COCl_2_ (10% in He) between 10 ml min^−1^ and 20 ml min^−1^ in 2.5 ml min^−1^ steps (actual flow of COCl_2_ between 1 ml min^−1^ and 2 ml min^−1^ in 0.25 ml min^−1^ steps), while varying the flow of the N_2_ diluent gas between 29 ml min^−1^ and 19 ml min^−1^ to maintain a total flow through the reactor of 69 ml min^−1^. The associated spectra are presented in Fig. S5.†

Fig. 1, 2 and 3 present the van't Hoff plots of COCl_2_ formation at 323 K, as a function of CO, Cl_2_ and COCl_2_ respectively. Table 1 presents the gradient of the slopes presented in these figures, with the errors representing the standard error in the linear fit of the five data-points recorded.

**Table 1:** Tabulated values of the slopes of the line of best fit presented in Fig. 1 (CO), Fig. 2 (Cl_2_) and Fig. 3 (COCl_2_)

Chemical species	CO	Cl_2_	COCl_2_
Gradient of slope	1.04 ± 0.02	0.46 ± 0.02	0.04 ± 0.01

With respect to the values presented in Table 1, the following experimentally determined rate law is shown:

2 *ν* = *k*[[CO]^1.04±0.02^[Cl_2_]^0.46±0.02^[COCl_2_]^0.04±0.01^]

With *ν* representing the rate of the reaction (mol COCl_2_ per min per g_(cat)_) and *k* representing a rate coefficient. Within experimental error, eqn (2) can be simplified to:

3 *ν* = *k*[CO]^1^[Cl_2_]^0.5^[COCl_2_]^0^

Thus, the relative order dependence of the reagents is described as first order with respect to CO, half order with respect to Cl_2_ and zero order with respect to COCl_2_. The simplified rate law (eqn (3)) is the same as was experimentally determined for phosgene synthesis over the Donau Supersorbon K40 formulation of activated carbon;^31^ thereby exhibiting consistency with the proposed reaction model for phosgene synthesis over the two industrial grade activated carbons.^33^

The findings are in alignment with the proposed mode of interaction in this reaction being molecular for CO (Scheme 1). With respect to the Langmuir adsorption isotherm,^37^ the half-order dependence on chlorine is indicative of dissociative adsorption of dichlorine, leading to adsorbed chlorine atoms.^25,26,30,31,33^ The zero-order dependence of the product^31^ indicates that, under the conditions of competitive adsorption explored in these rate measurements, the product of the reaction system does not influence its rate of production. The rate law depicted in eqn (3) avoids the complexity of combining Eley–Rideal and Langmuir–Hinshelwood kinetic expressions that Scheme 1 shows to be inherent to the reaction mechanism. Eqn (3) is also more transferable to the industrial scenario.^26^

### 3.2. Breakthrough measurements

An earlier examination of phosgene synthesis catalysis over Norit RX3 Extra^33^ showed comparable reactivity trends to that reported for Donau Supersorbon K40, an outcome that is consistent with the two substrates exhibiting comparable rate laws. However, certain distinctions between the performance of the two activated carbons was also noted: *viz.*, measurable differences in activation energies and differences in chlorine consumption profiles on thermal ramping.^33,36^ Indeed, with reference to Scheme 1, the latter was attributed to an equilibrium condition existing between active adsorbed chlorine atoms 
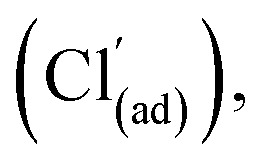
 and inactive adsorbed chlorine atoms 
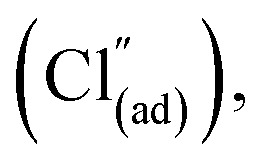
 as signified by eqn (4),4
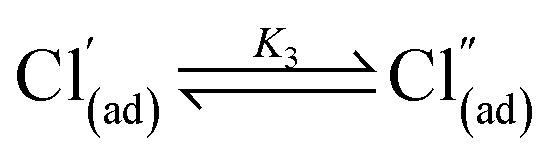
where *K*_3_ represents an equilibrium constant.^33^ Therefore, to further interrogate the system beyond the rate law dependence, a series of reagent/product breakthrough measurements were performed.

#### 3.2.1 Measurements at 323 K

**Fig. 4:**
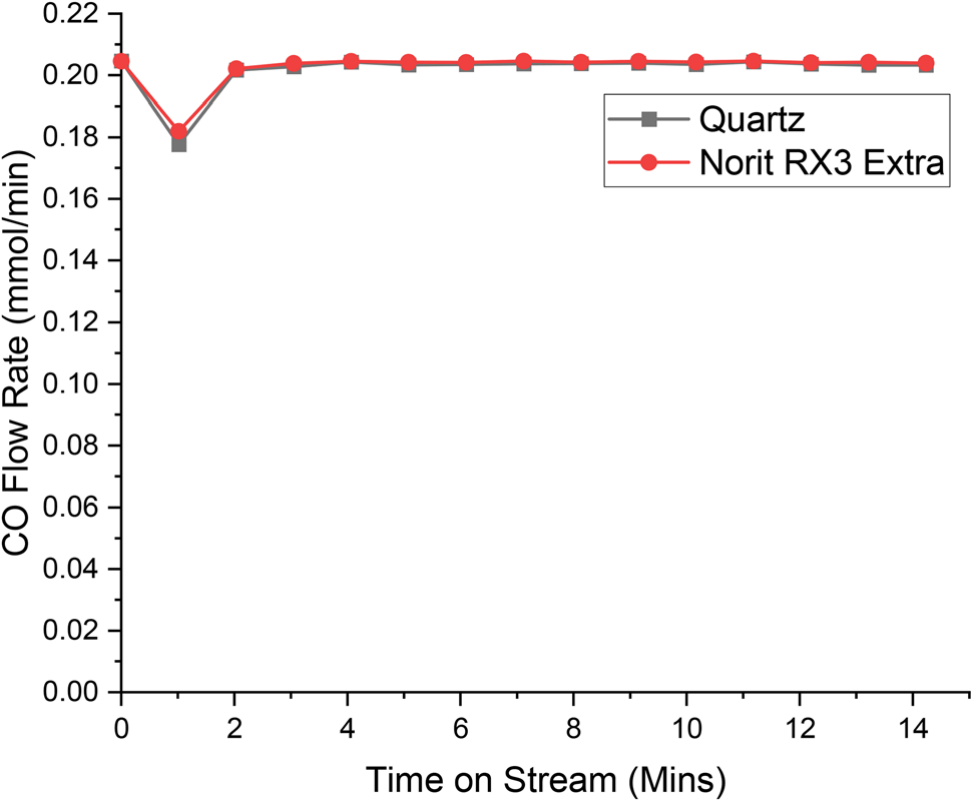
The response of directing a flow of 5 ml per min CO in 54 ml per min N_2_ over a reactor containing ground quartz (black) and the Norit RX3 Extra formulation of activated carbon (red), at 323 K. 0.4410 g of quartz was used, ground to between 250 and 500 μm. 0.1250 g of Norit RX3 Extra, ground to between 250 and 500 μm. The associated spectra are presented in Fig. S6 and S7.†

**Fig. 5:**
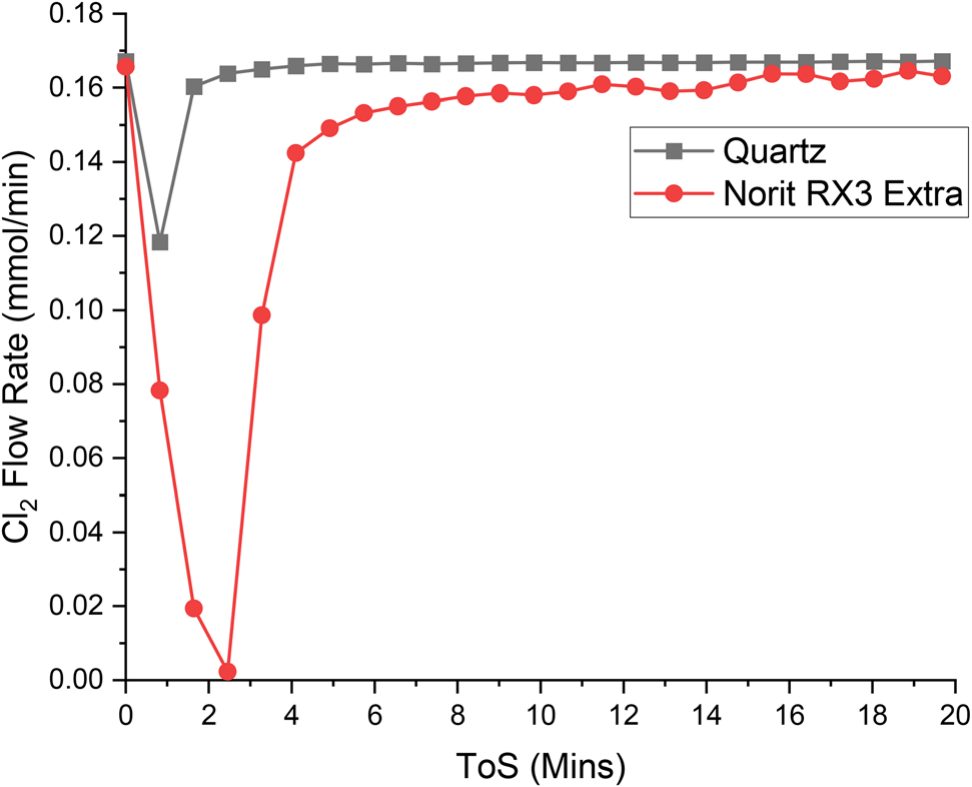
The response of directing a flow of 4 ml per min Cl_2_ in 55 ml per min N_2_ over a reactor containing ground quartz (black) and the Norit RX3 Extra formulation of activated carbon (red), at 323 K. 0.4410 g of quartz was used, ground to between 250 and 500 μm. 0.1250 g of Norit RX3 Extra, ground to between 250 and 500 μm. The associated spectra are presented in Fig. S8 and S9.†

**Fig. 6:**
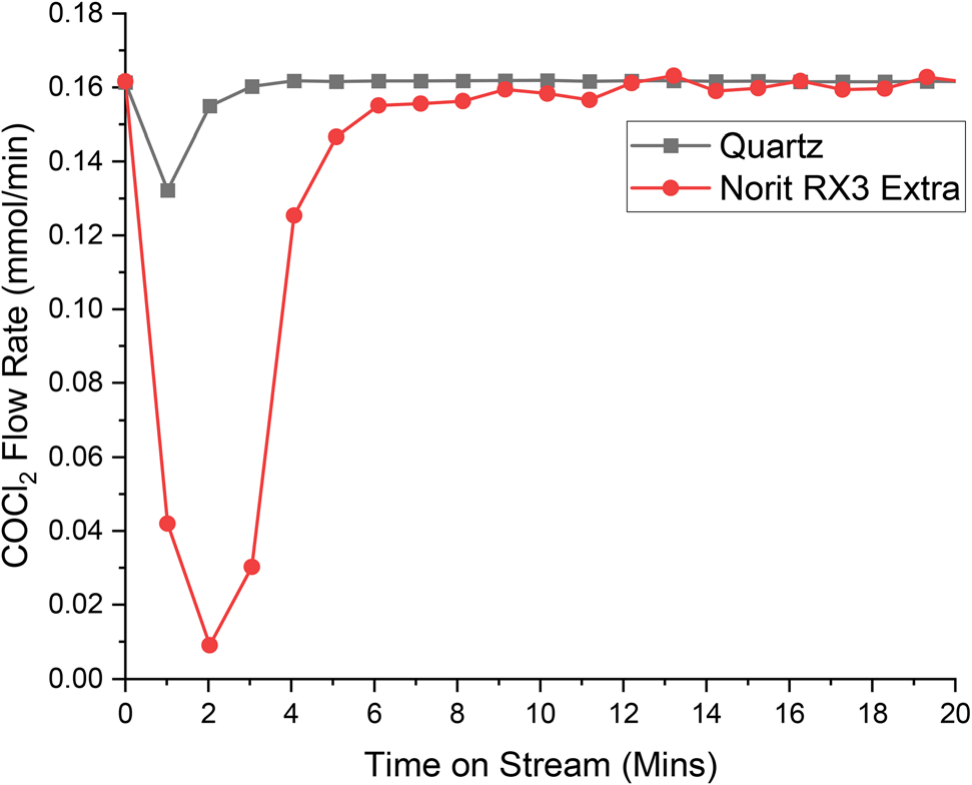
The response of directing a flow of 40 ml min^−1^ of 10% COCl_2_ in He (4 ml per min COCl_2_) and 19 ml per min N_2_ over a reactor containing ground quartz (black) and the Norit RX3 Extra formulation of activated carbon (red), at 323 K. 0.4410 g of quartz was used, ground to between 250 and 500 μm. 0.1250 g of Norit RX3 Extra, ground to between 250 and 500 μm. The associated spectra are presented in Fig. S12 and S13.†

Fig. 4, 5 and 6 present the respective breakthrough measurements for CO, Cl_2_ and COCl_2_ in relation to an inert analogue, quartz. As can be seen from all the figures, the quartz profiles show a feature for approximately 4 minutes time-on-stream (ToS). With reference to Fig. S2† and a previous publication,^30^ this feature is attributed to the switching of gas flow between the by-pass reactor and the reactor containing the catalyst. As such, calculation of the adsorption capacity of the catalyst for a given species will subtract the value attributed to this feature. The calculated adsorption capacities for the three chemical species are presented in Table 2.

**Table 2:** Tabulated values for the adsorption capacity of fresh Norit RX3 Extra for CO, Cl_2_ and COCl_2_. The integrated areas are presented in Table S4, ESI

Chemical species	CO	Cl_2_	COCl_2_
Adsorption capacity of Norit RX3 Extra (mMol g_(cat)_^−1^)	−0.056	3.52	3.92

With reference to Fig. 4, the adsorption characteristics of CO over Norit RX3 Extra and quartz are almost identical. Indeed, the CO adsorption over Norit RX3 Extra appears to be less than that of quartz, with Table 2 displaying a capacity of −0.056 mMol g_(cat)_^−1^ a reduction of 3.5% of the initial flow rate of 0.20 mMol CO per min. This difference is within the previously established experimental error of ±5.5%.^13^ As such, it is suggested that the adsorption characteristics of CO over Norit RX3 Extra are the same as that of quartz, *i.e.*, no adsorption of this chemical species occurs. This outcome replicates the CO dependency over Donau K40 (ref. 30) and is consistent with the suggestion that CO reacts ballistically with surface bound chlorine in an Eley–Rideal type mechanism,^32^ rather than CO being a surface bound species.

Chlorine and phosgene, on the other hand, exhibit significant adhesion to the catalyst, as shown in the breakthrough measurements presented in Fig. 5 and 6. The profile of adsorption of the two species are broadly similar, although differences are noted: COCl_2_ exhibits a larger initial adsorption that returns to baseline levels by 12 minutes ToS, whilst Cl_2_ initially shows a lowered adsorption with sustained adsorption evident until about 15 minutes ToS. The magnitude of the initial adsorption results in a larger adsorption capacity for COCl_2_ than Cl_2_, at 3.92 and 3.52 mMol g_(cat)_^−1^, respectively.

Table 2 indicates the following order of adsorption over the Norit RX3 Extra formulation of activated carbon: COCl_2_ > Cl_2_ ≫ CO, which can be interpreted in terms of adsorption coefficients as *K*_COCl_2__ > *K*_Cl_2__ ≫ *K*_CO_. This contrasts with the order over the Donau Supersorbon K40 formulation of activated carbon, where *K*_Cl_2__ > *K*_COCl_2__.^30^ Therefore, despite the similarities of the rate laws (Section 3.1) and not unsimilar reagent/product breakthrough characteristics, differences in the relative magnitudes of the materials' adsorption coefficients hints at differences in the active site distributions of the two formulations.

The finding of *K*_COCl_2__ > *K*_Cl_2__ over the Norit sample seemingly contradicts the observed zero-order dependence on phosgene as indicated by eqn (3). However, the contradiction is countenanced by recognising that the rate law measurements correspond to the catalyst operating in a competitive adsorption regime, whereas the breakthrough measurements constitute a non-competitive process. Further work is required to better understand this phenomenon. Nonetheless, collectively, the rate law and breakthrough measurements suggest that in an environment of CO, Cl_2_, COCl_2_ and N_2_ the active site(s) for phosgene synthesis may be exclusive for chlorine adsorption.

#### 3.2.2 Thermal treatments

**Fig. 7:**
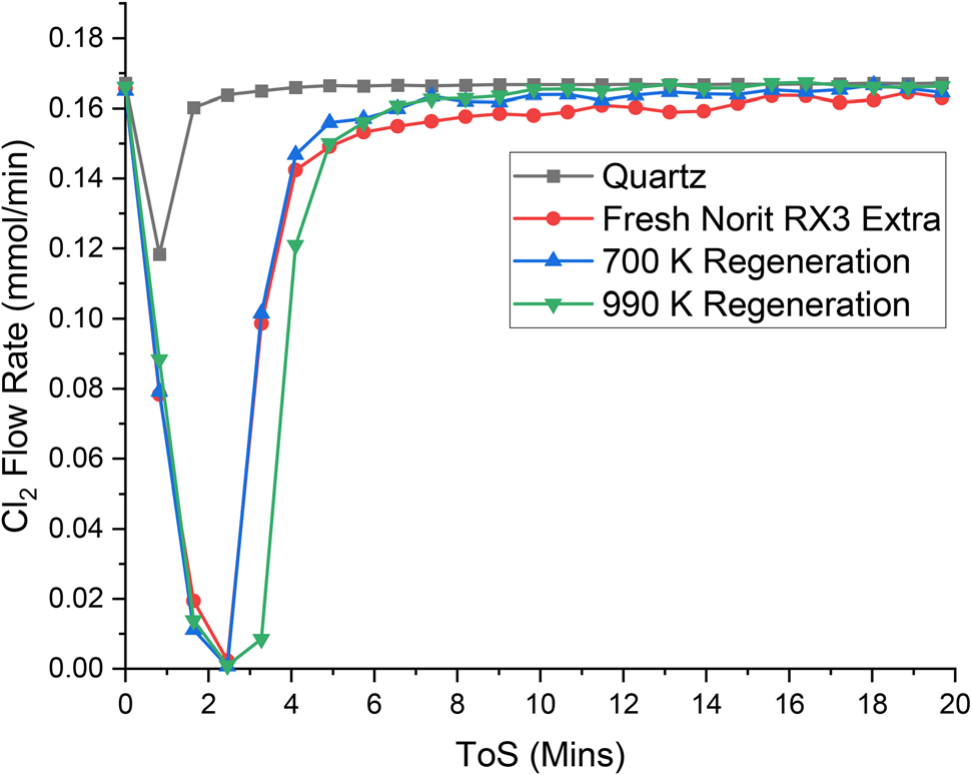
The response of directing a flow of 4 ml per min Cl_2_ in 55 ml per min N_2_ over a reactor containing ground quartz (black) and the Norit RX3 Extra formulation of activated carbon (red), at 323 K. 0.4410 g of quartz was used, ground to between 250 and 500 μm. 0.1250 g of Norit RX3 Extra, ground to between 250 and 500 μm. The same sample of Norit RX3 Extra, underwent a post-chlorination temperature ramp to 700 K at a ramp rate of 5 K min^−1^ (10 ml per min N_2_) was then re-dosed with chlorine under the same conditions (blue). Finally, the same sample underwent a further thermal treatment to 990 K under the same conditions and was then re-dosed with chlorine under the same conditions (green). The associated spectra are presented in Fig. S8–S11.†

To further investigate contributions from the aforementioned type-I (phosgene synthesis active) and type-II (phosgene synthesis inactive) chlorine adsorption sites, thermal ramping followed by chlorine re-adsorption studies were performed, as described in Section 2.2.2. Fig. 7 presents the chlorine breakthrough profiles for the original 323 K measurement (red circles) alongside profiles for chlorine retention at 323 K after thermal ramping to 700 (blue up-triangles, below the onset thermal decomposition temperature, as previously reported by TGA in a 2% O_2_/Ar mixture^33^) and 990 K (green down-triangles, above the onset thermal decomposition temperature^33^); Table 3 presents the associated adsorption capacities.

**Table 3:** Tabulated values for the Cl_2_ adsorption capacity of fresh Norit RX3 Extra, the same sample of Norit RX3 Extra, which post-chlorination underwent a temperature ramp to 700 K at a ramp rate of 5 K min^−1^ (10 ml per min N_2_) and the same sample which underwent a further thermal treatment to 990 K under the same conditions. The integrated areas are presented in Table S4, ESI

Catalyst conditions	Fresh	700 K treatment	990 K treatment
Chlorine adsorption (mMol g_(cat)_^−1^)	3.52	3.20	3.92

Previous studies of Donau Supersorbon K40 (ref. 30) utilised a thermal treatment of 673 K to signify the presence of type-I and type-II chlorine adsorption sites, where the chlorine retained at type-II sites possessed a binding strength of a magnitude that it could not be removed by thermal treatment at 673 K; chlorine adsorbed over type-I functionalities being removed *via* the thermal treatment. Re-dosing the catalyst with chlorine on completion of a thermal treatment stage re-populated the type-I sites. By this reasoning, approximately 35% of the total chlorine adsorption sites of Donau Supersorbon K40 were attributed to type-II sites.^30^

In contrast, based on outcomes presented in Fig. 7 and Table 4 shows only 10% (0.32 mMol chlorine per g_(cat)_) of the total Cl_2_ adsorption sites of Norit RX3 Extra are type-II sites. Thus, the proportion of type-II sites between the two catalysts is 3.5 times higher for Donau Supersorbon K40 than Norit RX3 Extra. This is broadly consistent with mass balance profiles showing the chlorine retained by Donau Supersorbon K40 to be 5.5 times higher than that of Norit RX3 Extra.^31,33^ Furthermore, post-reaction EDX analysis of both catalysts^30,33^ indicates the retained chlorine present is 2.2 times higher over the Donau material than the Norit.^30^ Consideration of the factors outlined above, shows the number of type-II Cl_2_ adsorption sites present on Donau Supersorbon K40 to be higher than those that are accessible on Norit RX3 Extra.

**Table 4:** Values for the chlorine adsorption capacity of the type-I and type-II chlorine adsorption sites

Chlorine adsorption site	Type-I	Type-II
Chlorine capacity (mMol g_(cat)_^−1^)	3.2	0.32

Thus, Norit RX3 Extra possesses between 3.5 and 5.5 times less type-II chlorine adsorption sites than Donau Supersorbon K40, as estimated by the chlorine capacities determined by thermal treatment and steady-state chlorine retention values, respectively. This discrepancy in how chlorine partitions between the active sites present on both carbons may be linked to catalytic performance. Specifically, under broadly comparable steady-state conditions at 323 K the Norit RX3 Extra phosgene production rate is approximately twice that observed for Donau Supersorbon K40 (0.33 mMol min^−1^ g_(cat)_^−1^ (ref. 33) *cf.* 0.17 mMol COCl_2_ per min per g_(cat)_ (ref. 31)). This comparison meshes with outcomes from Mitchell and co-workers who reported Norit RX3 Extra to exhibit superior COCl_2_ formation rates compared to Donau Supersorbon K40.^3^ Thus, the greater accessibility of type I sites for the Norit carbon at 323 K leads to enhanced phosgene formation rates compared to the Donau material, due the former possessing a greater density of type-I sites.^31,33^

It was originally envisaged that heating the sample above the onset decomposition temperature would lead to a degree of surface annealing and, consequently, a reduction in the active site density presented by the material. Interestingly, inspection of the high temperature regeneration in Fig. 7 and Table 3 shows that, compared to the fresh sample, the procedure actually leads to increased chlorine capacity (3.92 mMol g_cat_^−1^ compared to 3.52 mMol g_cat_^−1^). It is possible that the origin of this occurrence may be *via* a thermally induced chemical etching of the catalyst surface, possibly in a similar manner to that reported for the etching of highly ordered graphitic crystallites found throughout the structure of activated carbons,^17^ which can lead to defects in the exposed graphitic sheets.^38–40^ These defects are thought to be electron rich in nature^40^ and, therefore, an electrophile such as chlorine would be amenable to adsorption at such a defect. Therefore, when the etched surface is re-dosed with chlorine it is tentatively suggested that this could lead to an increased chlorine adsorption capacity, as evidenced in Fig. 7. Further work is required to better understand this technically important issue.

## 4. Conclusions

Examination of the performance of Norit RX3 Extra in a role as a phosgene synthesis catalyst has been examined within the context of a previously proposed reaction model and the following points are noted.

• The kinetic behaviour of the synthesis of COCl_2_ from CO and Cl_2_ over the Norit RX3 Extra formulation of activated carbon was found to conform to a previously reported reaction model (Scheme 1) and exhibits a near identical rate law to that reported for an alternative activated carbon formulation (Donau Supersorbon K40). The rate law for COCl_2_ synthesis over the two industrial grade activated carbon catalysts examined is as follows: first order with respect to CO, half order with respect to Cl_2_ and zero order with respect to COCl_2_.

• Analysis of non-competitive adsorption studies over fresh catalyst show minimal CO adsorption, consistent with an Eley–Rideal type step in the synthesis process, whilst Cl_2_ and COCl_2_ adsorb readily.

• The addition of a thermal regeneration treatment in chlorine breakthrough measurements shows Norit RX3 Extra to possess a higher proportion of active chlorine adsorption sites compared to Donau Supersorbon K40. This observation correlates with the higher phosgene formation rate observed over Norit RX3 Extra.

• Thermal cycling of chlorine dosed samples past the thermal decomposition temperature (as identified by TGA), suggests that etching of the catalyst occurs due to thermal and oxidative stress.

• Broad similarities with small differences between the performance of the Norit RX3 Extra and Donau Supersorbon K40 samples appear to validate the generic applicability of the previously postulated reaction model, whilst simultaneously establishing differences in the active site distributions of the two activated carbons.

## Conflicts of interest

There are no conflicts of interest to declare.

## Supplementary Material

RA-015-D5RA04045K-s001

## Data Availability

Datasets for the article are available at the University of Glasgow Library *via* DOI: https://doi.org/10.5525/gla.researchdata.1987.
