# Supplementary material for: Phosgene synthesis catalysis: reaction kinetics and adsorption characteristics over Norit RX3 Extra activated carbon formulation
Source: RSC Adv. 2025 Jul 16;15(31):25178–86. doi: 10.1039/d5ra04045k (PMC12264745; doi:10.1039/d5ra04045k)
Supplement: RA-015-D5RA04045K-s001 [file RA-015-D5RA04045K-s001.pdf]

Supplementary Information (SI) for RSC Advances.
This journal is © The Royal Society of Chemistry 2025

Supporting Information

Phosgene synthesis catalysis: reaction kinetics and adsorption characteristics over the Norit RX3 Extra activated carbon formulation

Rory Hughes and David Lennon*

School of Chemistry, Joseph Black Building, University of Glasgow, Glasgow, G12 8QQ, UK.

Table S1: Tabulated flow rates for CO, Cl_2 and N_2 (partitioned as pre- and post-reactor flows) used in the determination of the CO order dependence of the phosgene synthesis reaction.

Table S2: Tabulated flow rates for CO, Cl_2 and N_2 (partitioned as pre- and post-reactor flows) used in the determination of the Cl_2 order dependence of the phosgene synthesis reaction.

Figure S1: Line diagram of experimental apparatus showing modifications to include an additional nitrogen mass flow controller, highlighted in red.

Table S3: Tabulated flow rates for CO, Cl_2, $COCl_2$ and N_2 (partitioned as pre- and post-reactor flows) used in the determination of the $COCl_2$ order dependence of the phosgene synthesis reaction.

Figure S2: A plot of the negative CO flow rate of the response of directing a flow of 5 ml/min CO in 54 ml/min N_2 over a reactor containing the Norit RX3 Extra formulation of activated carbon, at 323 K. 0.1250 g of Norit RX3 Extra, ground to between 250 and 500 µm. The plot shows the region which was integrated to produce an area.

Figure S3: Selected IR spectra of the concentration dependence of CO for the reaction between CO and Cl_2 over 0.1250 g of the Norit RX3 Extra formulation of activated carbon, ground to between 250 and 500 µm at 323 K. The order dependence was determined by fixing Cl_2 at 15 ml/min and varying the flow rate CO between 2 ml/min and 6 ml/min in 1 ml/min steps, while varying the flow of the N_2 diluent gas between 42 ml/min and 38 ml/min to maintain a total flow through the reactor of 59 ml/min.

Figure S4: Selected UV spectra of the concentration dependence of Cl_2 for the reaction between CO and Cl_2 over 0.1250 g of the Norit RX3 Extra formulation of activated carbon, ground to between 250 and 500 µm at 323 K. The order dependence was determined by fixing CO at 15 ml/min and varying the flow rate CO between 6 ml/min and 10 ml/min in 1 ml/min steps, while varying the flow of the N_2 diluent gas between 48 ml/min and 44 ml/min to maintain a total flow through the reactor of 69 ml/min.

Figure S5: Selected IR spectra of the concentration dependence of $COCl_2$ for the reaction between CO and Cl_2 over 0.1250 g of the Norit RX3 Extra formulation of activated carbon, ground to between 250 and 500 µm at 323 K. The order dependence was determined by fixing CO and Cl_2 at 15 ml/min and varying the flow rate $COCl_2$ (10 % in He) between 10 ml/min and 20 ml/min in 2.5 ml/min steps (actual flow of $COCl_2$ between 1 ml/min and 2 ml/min in 0.25 ml/min steps), while varying the flow of the N_2 diluent gas between 29 ml/min and 19 ml/min to maintain a total flow through the reactor of 69 ml/min.

Figure S6: Selected IR spectra of the response of directing a flow of 5 ml/min CO in 54 ml/min N_2 over a reactor containing ground quartz at 323 K. 0.4410 g of quartz was used, ground to between 250 and 500 µm.

Figure S7: The response of directing a flow of 5 ml/min CO in 54 ml/min N_2 over a reactor containing the Norit RX3 Extra formulation of activated carbon, at 323 K. 0.1250 g of Norit RX3 Extra, ground to between 250 and 500 µm.

Figure S8: Selected UV Spectra of the response of directing a flow of 4 ml/min Cl_2 in 55 ml/min N_2 over a reactor containing ground quartz at 323 K. 0.4410 g of quartz was used, ground to between 250 and 500 µm.

Figure S9: Selected UV Spectra of the response of directing a flow of 4 ml/min Cl_2 in 55 ml/min N_2 over a reactor containing the Norit RX3 Extra formulation of activated carbon, at 323 K. 0.1250 g of Norit RX3 Extra, ground to between 250 and 500 µm.

Figure S10: Selected UV spectra of the response of directing a flow of 4 ml/min Cl_2 in 55 ml/min N_2 over a reactor containing a previously chlorinated sample of Norit RX3 Extra, that underwent a post-chlorination temperature ramp to 700 K at a ramp rate of 5 K/min 10 ml/min N_2 was then re-dosed with chlorine under a flow of 4 ml/min Cl_2 in 55 ml/min N_2 over a reactor containing ground quartz at 323 K.

Figure S11:Selected UV spectra of the response of directing a flow of 4 ml/min Cl_2 in 55 ml/min N_2 over a reactor containing a previously chlorinated sample of Norit RX3 Extra, that underwent chlorination and a post-chlorination temperature ramp to 700 K at a ramp rate of 5 K/min 10 ml/min N_2 This sample was then thermally treated by temperature ramp to 990 K at a ramp rate of 5 K/min 10 ml/min N_2 re-dosed with chlorine under a flow of 4 ml/min Cl_2 in 55 ml/min N_2 over a reactor containing ground quartz at 323 K.

Figure S12: Selected IR Spectra of the response of directing a flow of 40 ml/min of 10 % $COCl_2$ in He (4 ml/min $COCl_2$) and 19 ml/min N_2 over a reactor containing ground quartz at 323 K. 0.4410 g of quartz was used, ground to between 250 and 500 µm.

Figure S13: Selected IR Spectra of the response of directing a flow of 40 ml/min of 10 % $COCl_2$ in He (4 ml/min $COCl_2$) and 19 ml/min N_2 over a reactor containing the Norit RX3 Extra formulation of activated carbon, at 323 K. 0.1250 g of Norit RX3 Extra, ground to between 250 and 500 µm.

Table S4: Tabulated areas produced from integrating the response of flowing CO, Cl_2 and $COCl_2$ over quartz, Norit RX3 Extra and thermally treated Norit RX3 Extra.

Sample	CO Flow Rate (ml/min)	Cl_2 Flow Rate (ml/min)	Pre-Reactor N_2 Flow Rate (ml/min)	Post Reactor N_2 Flow Rate (ml/min)
1	2	15	42	100
2	3	15	41	100
3	4	15	40	100
4	5	15	39	100
5	6	15	38	100

Table S1: Tabulated flow rates for CO, Cl_2 and N_2 (partitioned as pre- and post-reactor flows) used in the determination of the CO order dependence of the phosgene synthesis reaction.

Sample	CO Flow Rate (ml/min)	Cl_2 Flow Rate (ml/min)	Pre-Reactor N_2 Flow Rate (ml/min)	Post Reactor N_2 Flow Rate (ml/min)
1	15	6	48	90
2	15	7	47	90
3	15	8	46	90
4	15	9	45	90
5	15	10	44	90

Table S2: Tabulated flow rates for CO, Cl_2 and N_2 (partitioned as pre- and post-reactor flows) used in the determination of the Cl_2 order dependence of the phosgene synthesis reaction.

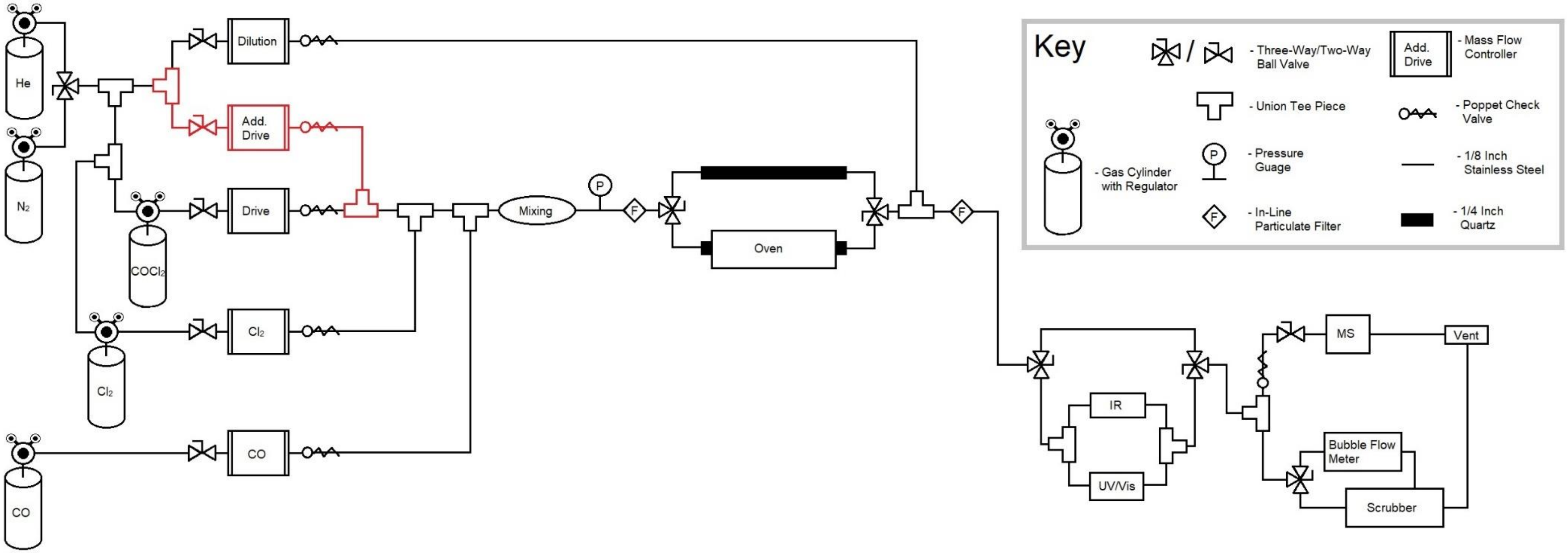

<u>Figure S1</u>: Line diagram of experimental apparatus showing modifications to include an additional nitrogen mass flow controller, highlighted in red.

Sample	CO Flow Rate (ml/min)	Cl_2 Flow Rate (ml/min)	10% $COCl_2$ in He Flow Rate (ml/min)	Pre-Reactor N_2 Flow Rate (ml/min)	Post Reactor N_2 Flow Rate (ml/min)
1	15	15	10	29	90
2	15	15	12.5	26.5	90
3	15	15	15	24	90
4	15	15	17.5	21.5	90
5	15	15	20	19	90

Table S3: Tabulated flow rates for CO, Cl_2, $COCl_2$ and N_2 (partitioned as pre- and post-reactor flows) used in the determination of the $COCl_2$ order dependence of the phosgene synthesis reaction.

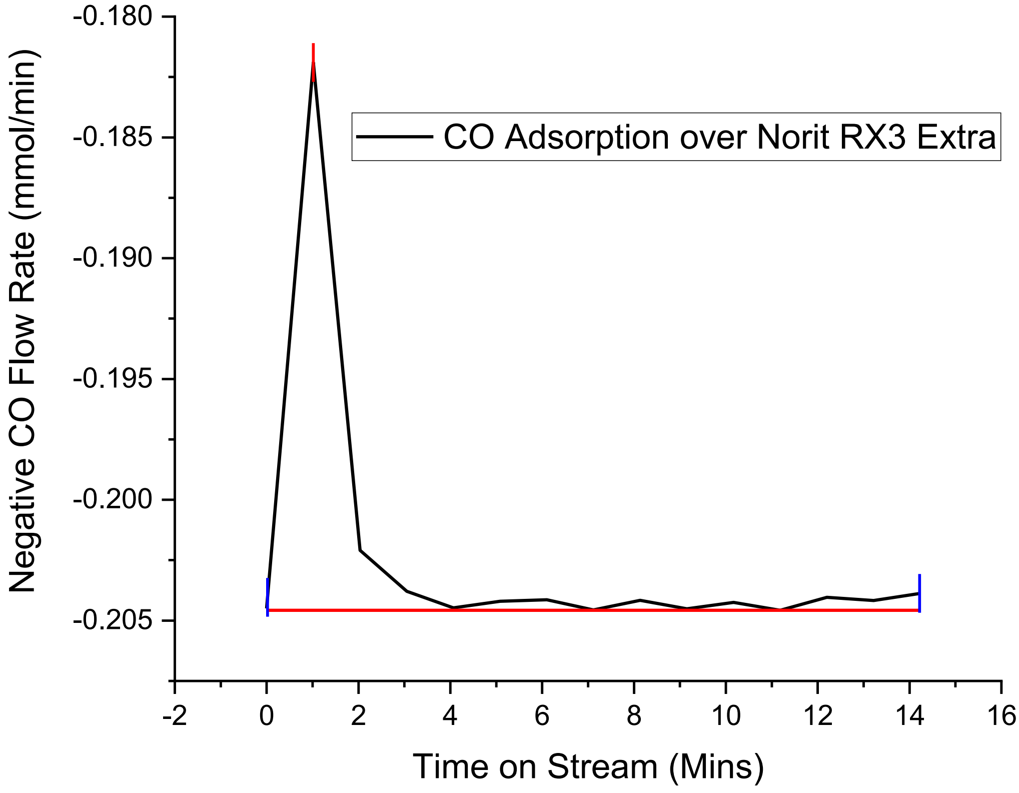

Figure S2: A plot of the negative CO flow rate of the response of directing a flow of 5 ml/min CO in 54 ml/min N_2 over a reactor containing the Norit RX3 Extra formulation of activated carbon, at 323 K. 0.1250 g of Norit RX3 Extra, ground to between 250 and 500 µm. The plot shows the region which was integrated to produce an area.

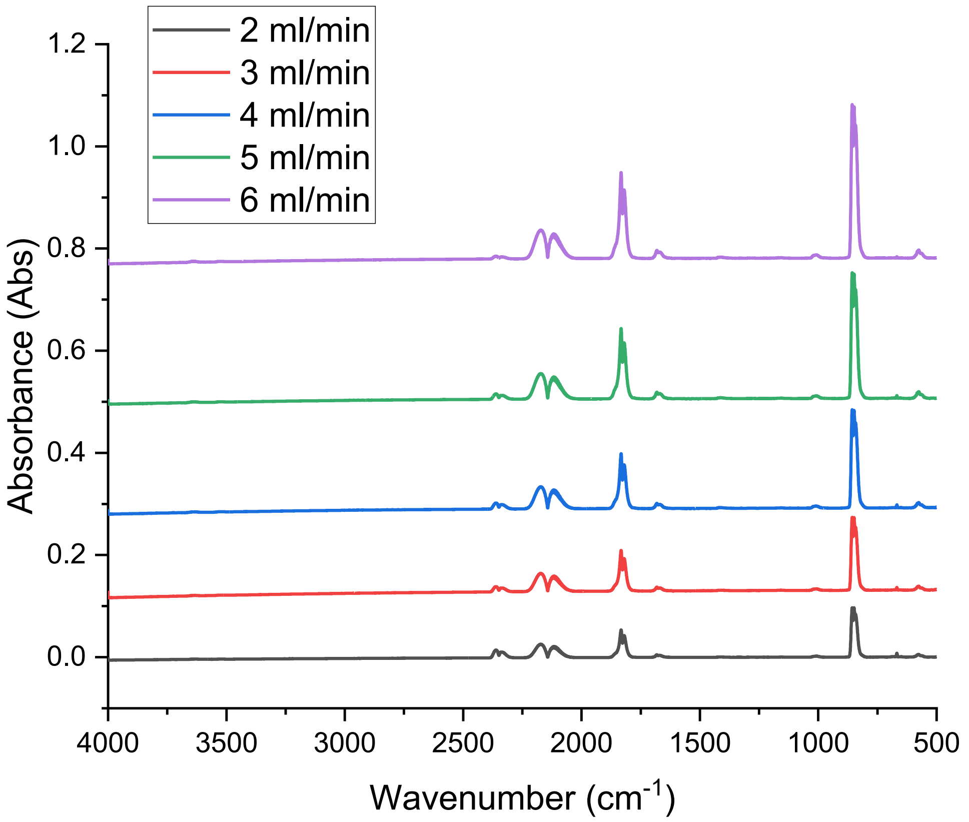

Figure S3: Selected IR spectra of the concentration dependence of CO for the reaction between CO and Cl_2 over 0.1250 g of the Norit RX3 Extra formulation of activated carbon, ground to between 250 and 500 µm at 323 K. The order dependence was determined by fixing Cl_2 at 15 ml/min and varying the flow rate CO between 2 ml/min and 6 ml/min in 1 ml/min steps, while varying the flow of the N_2 diluent gas between 42 ml/min and 38 ml/min to maintain a total flow through the reactor of 59 ml/min.

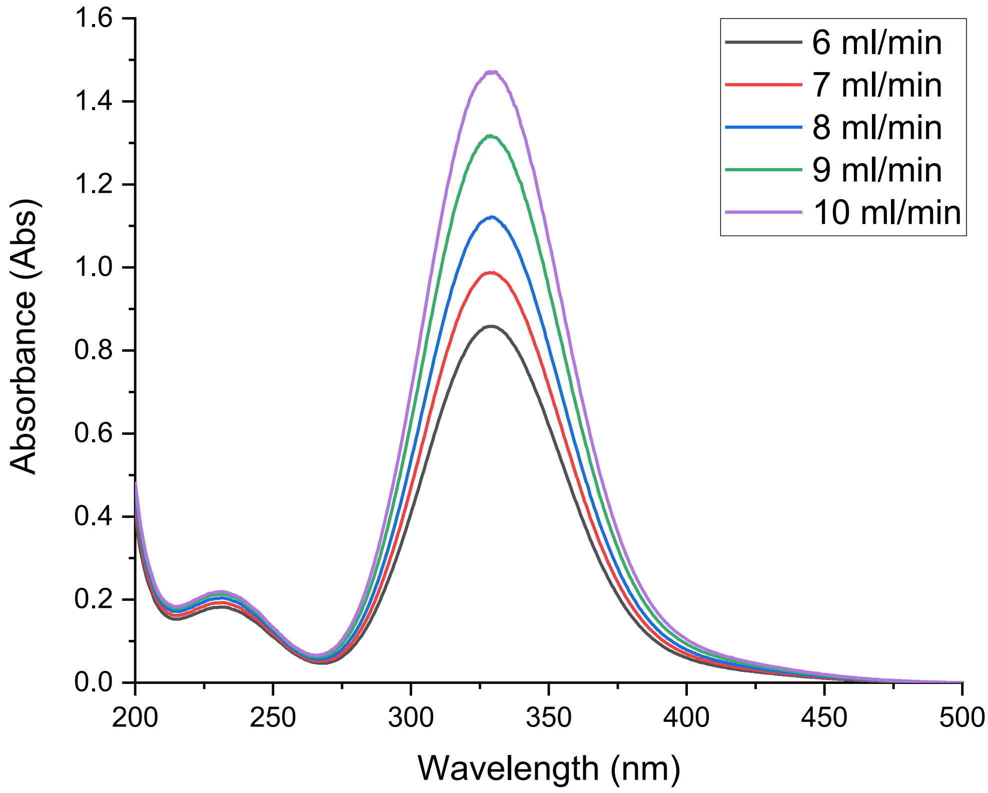

Figure S4: Selected UV spectra of the concentration dependence of Cl_2 for the reaction between CO and Cl_2 over 0.1250 g of the Norit RX3 Extra formulation of activated carbon, ground to between 250 and 500 μm at 323 K. The order dependence was determined by fixing CO at 15 ml/min and varying the flow rate CO between 6 ml/min and 10 ml/min in 1 ml/min steps, while varying the flow of the N_2 diluent gas between 48 ml/min and 44 ml/min to maintain a total flow through the reactor of 69 ml/min.

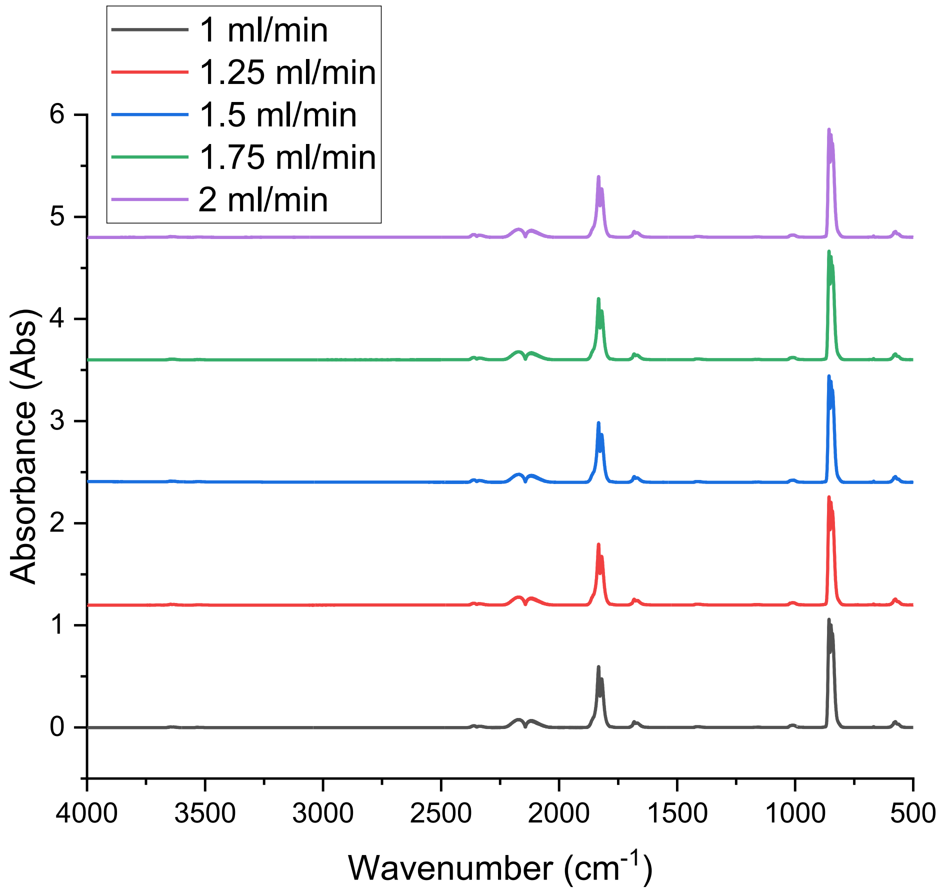

Figure S5: Selected IR spectra of the concentration dependence of $COCl_2$ for the reaction between CO and Cl_2 over 0.1250 g of the Norit RX3 Extra formulation of activated carbon, ground to between 250 and 500 µm at 323 K. The order dependence was determined by fixing CO and Cl_2 at 15 ml/min and varying the flow rate $COCl_2$ (10 % in He) between 10 ml/min and 20 ml/min in 2.5 ml/min steps (actual flow of $COCl_2$ between 1 ml/min and 2 ml/min in 0.25 ml/min steps), while varying the flow of the N_2 diluent gas between 29 ml/min and 19 ml/min to maintain a total flow through the reactor of 69 ml/min.

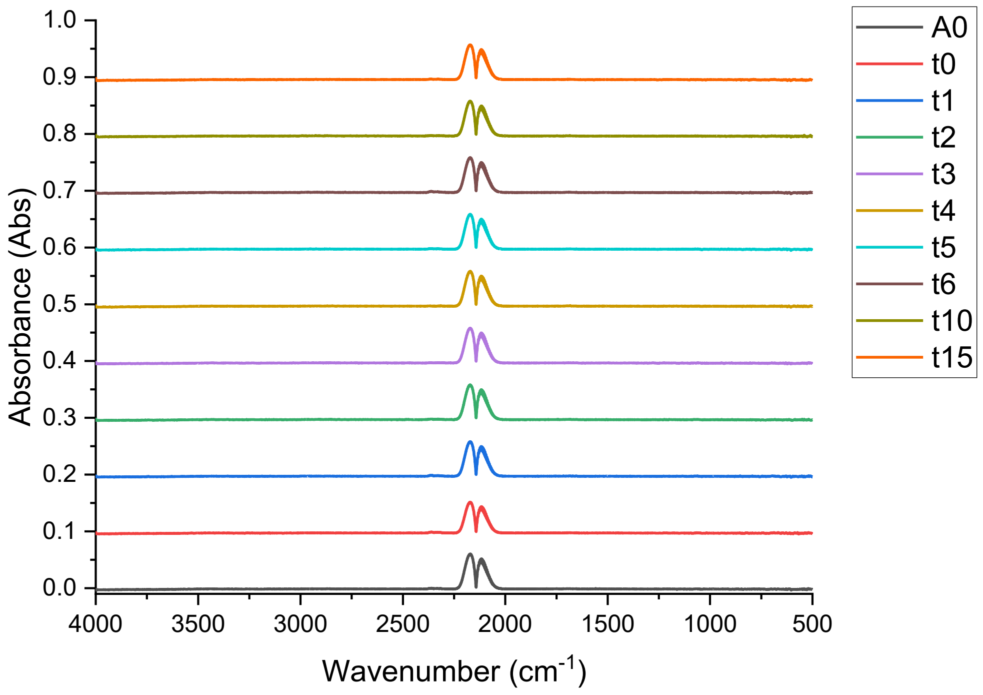

Figure S6: Selected IR spectra of the response of directing a flow of 5 ml/min CO in 54 ml/min N_2 over a reactor containing ground quartz at 323 K. 0.4410 g of quartz was used, ground to between 250 and 500 µm.

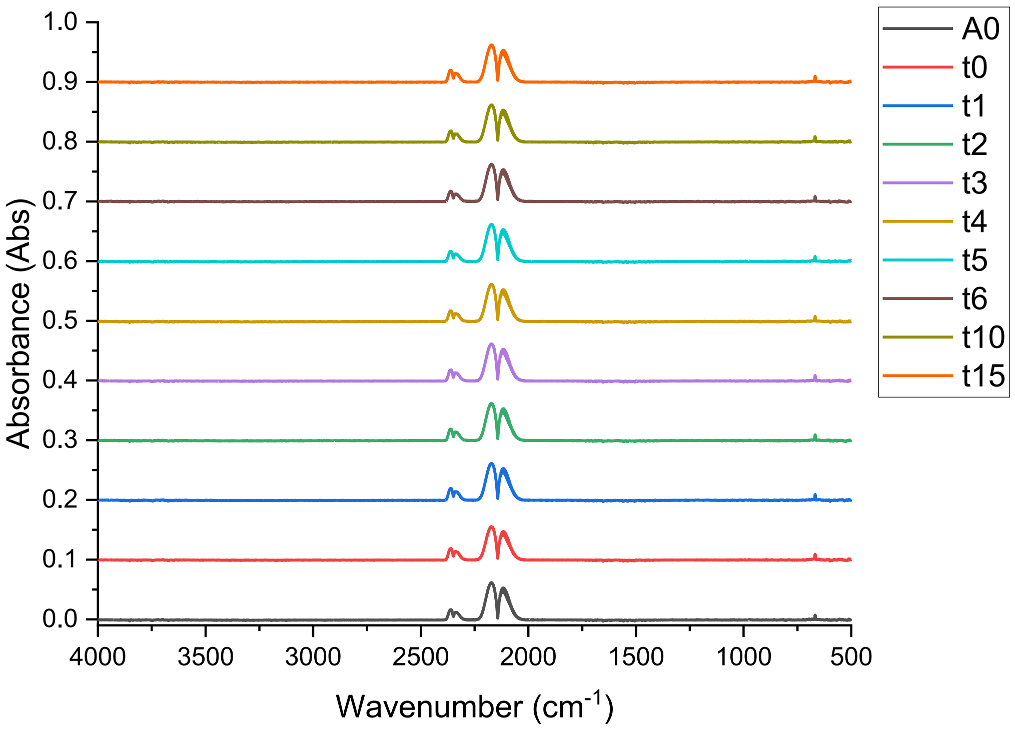

Figure S7: The response of directing a flow of 5 ml/min CO in 54 ml/min N_2 over a reactor containing the Norit RX3 Extra formulation of activated carbon, at 323 K. 0.1250 g of Norit RX3 Extra, ground to between 250 and 500 µm.

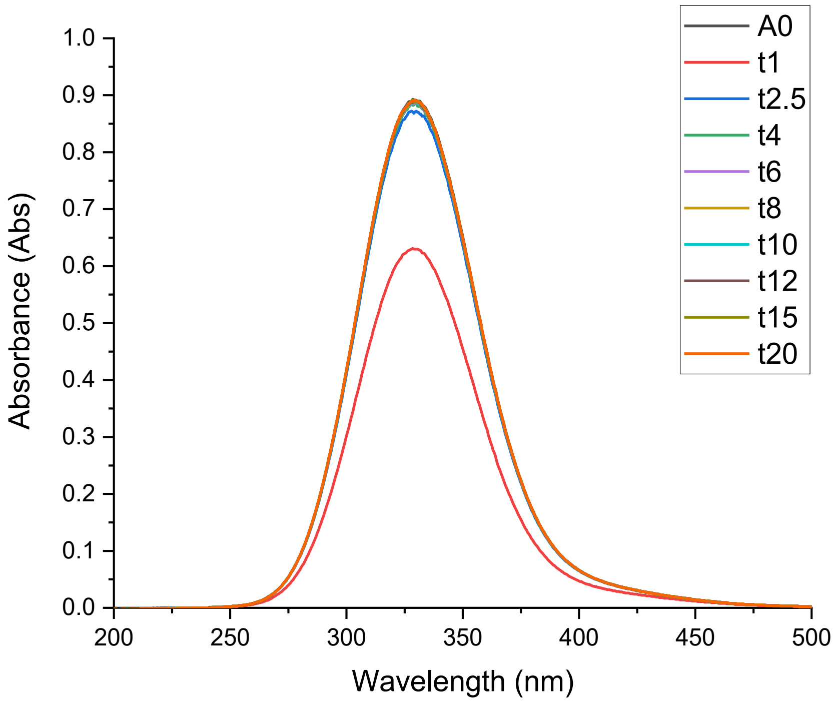

Figure S8: Selected UV Spectra of the response of directing a flow of 4 ml/min Cl_2 in 55 ml/min N_2 over a reactor containing ground quartz at 323 K. 0.4410 g of quartz was used, ground to between 250 and 500 µm.

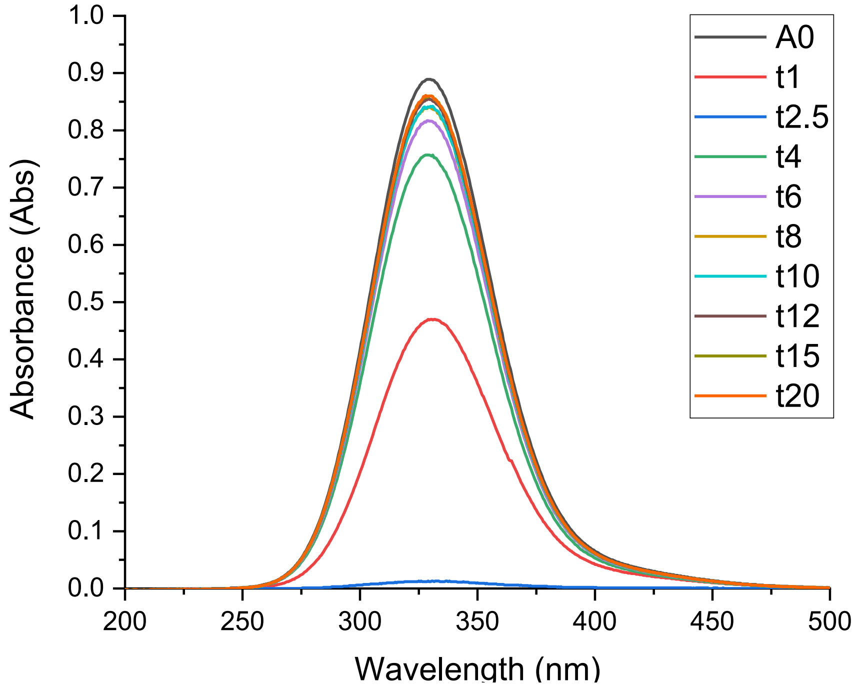

Figure S9: Selected UV Spectra of the response of directing a flow of 4 ml/min Cl_2 in 55 ml/min N_2 over a reactor containing the Norit RX3 Extra formulation of activated carbon, at 323 K. 0.1250 g of Norit RX3 Extra, ground to between 250 and 500 µm.

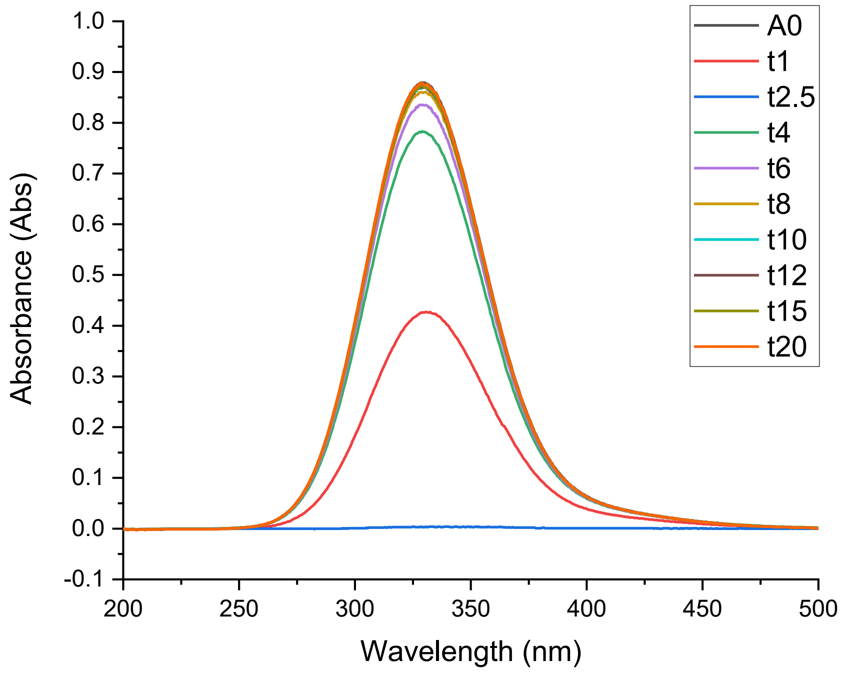

Figure S10: Selected UV spectra of the response of directing a flow of 4 ml/min Cl_2 in 55 ml/min N_2 over a reactor containing a previously chlorinated sample of Norit RX3 Extra, that underwent a post-chlorination temperature ramp to 700 K at a ramp rate of 5 K/min 10 ml/min N_2 was then re-dosed with chlorine under a flow of 4 ml/min Cl_2 in 55 ml/min N_2 over a reactor containing ground quartz at 323 K.

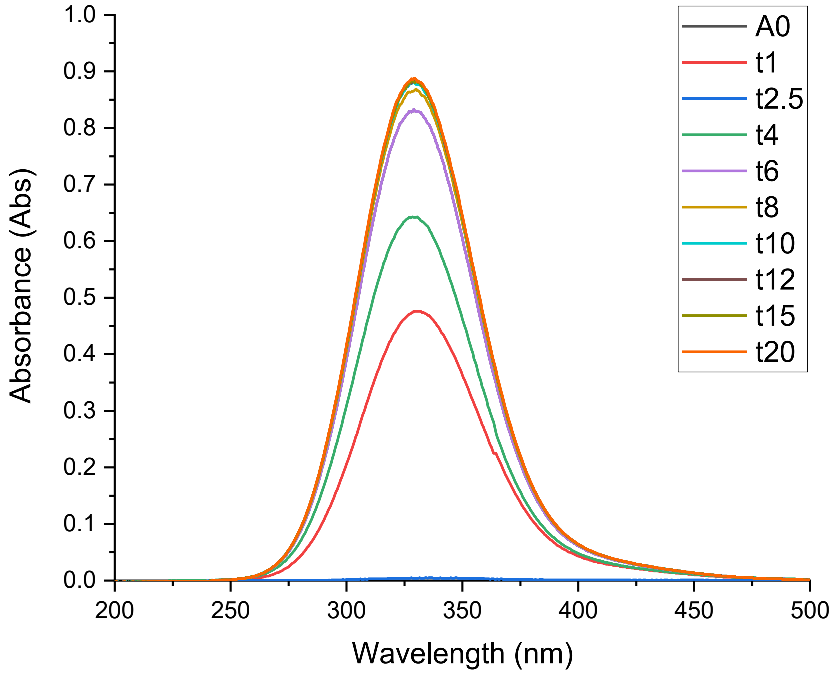

Figure S11:Selected UV spectra of the response of directing a flow of 4 ml/min Cl_2 in 55 ml/min N_2 over a reactor containing a previously chlorinated sample of Norit RX3 Extra, that underwent chlorination and a post-chlorination temperature ramp to 700 K at a ramp rate of 5 K/min 10 ml/min N_2 This sample was then thermally treated by temperature ramp to 990 K at a ramp rate of 5 K/min 10 ml/min N_2 re-dosed with chlorine under a flow of 4 ml/min Cl_2 in 55 ml/min N_2 over a reactor containing ground quartz at 323 K.

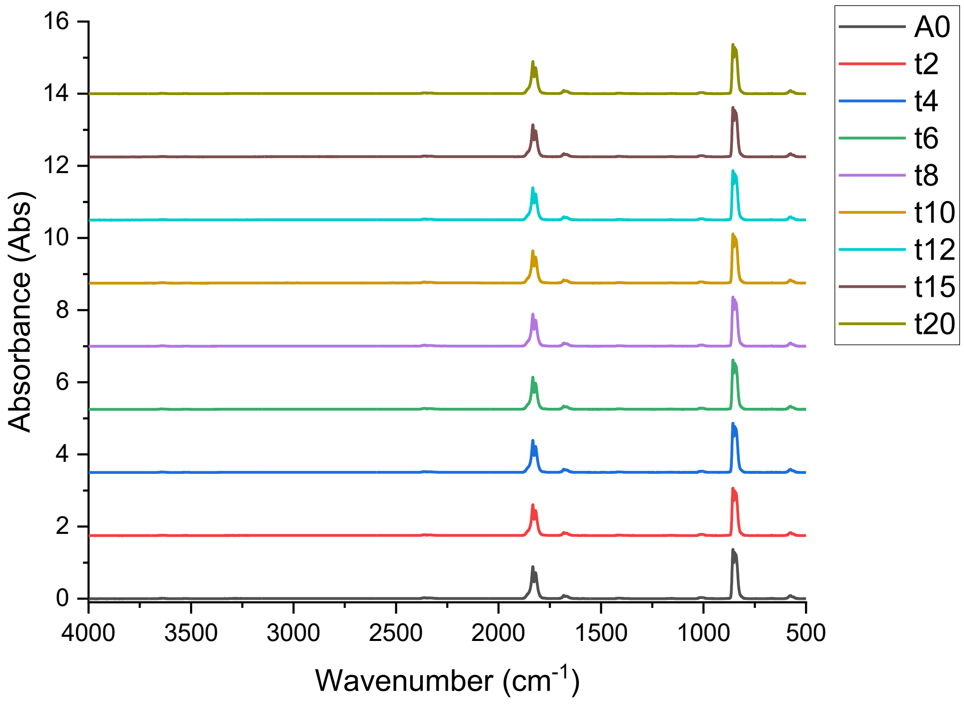

Figure S12: Selected IR Spectra of the response of directing a flow of 40 ml/min of 10 % $COCl_2$ in He (4 ml/min $COCl_2$) and 19 ml/min N_2 over a reactor containing ground quartz at 323 K. 0.4410 g of quartz was used, ground to between 250 and 500 µm.

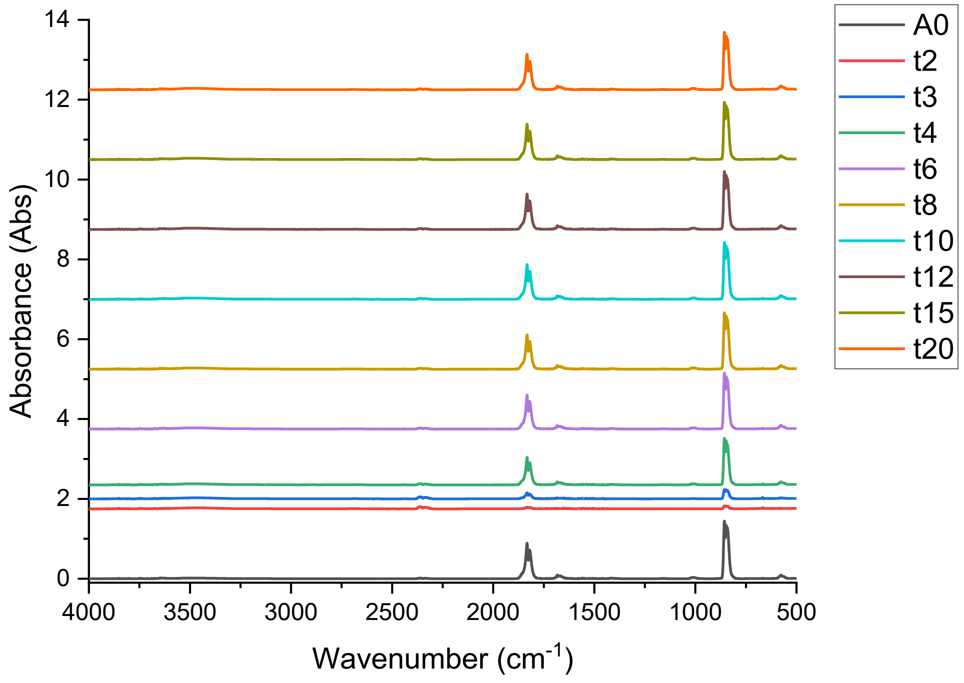

Figure S13: Selected IR Spectra of the response of directing a flow of 40 ml/min of 10 % $COCl_2$ in He (4 ml/min $COCl_2$) and 19 ml/min N_2 over a reactor containing the Norit RX3 Extra formulation of activated carbon, at 323 K. 0.1250 g of Norit RX3 Extra, ground to between 250 and 500 µm.

Sample	Integrated Area (mmol/min)
CO – Quartz	0.03639
CO – Norit RX3 Extra	0.02947
Cl_2 – Quartz	0.05836
Cl_2 – Norit RX3 Extra (Fresh)	0.49925
Cl_2 – Norit RX3 Extra (700 K Regen)	0.46068
Cl_2 – Norit RX3 Extra (990 K Regen)	0.54929
$COCl_2$ – Quartz	0.04264
$COCl_2$ – Norit RX3 Extra	0.52918

Table S4: Tabulated areas produced from integrating the response of flowing CO, Cl_2 and $COCl_2$ over quartz, Norit RX3 Extra and thermally treated Norit RX3 Extra.
